# VISMapper: ultra-fast exhaustive cartography of viral insertion sites for gene therapy

**DOI:** 10.1186/s12859-017-1837-z

**Published:** 2017-09-20

**Authors:** José M. Juanes, Asunción Gallego, Joaquín Tárraga, Felipe J. Chaves, Pablo Marín-Garcia, Ignacio Medina, Vicente Arnau, Joaquín Dopazo

**Affiliations:** 1 Departamento de Informática, Escuela Técnica Superior de Ingeniería (ETSE), Universidad de Valencia, 46100 Valencia, Burjassot Spain; 2 Computational Genomics Department, Prince Felipe Research Center, 46012 Valencia, Spain; 3 Clinical Bioinformatics Research Area, Fundación Progreso y Salud, Hospital Virgen del Rocío, 41013 Sevilla, Spain; 4 Bioinformatics in Rare Diseases (BiER), Centro de Investigación Biomédica en Red de Enfermedades Raras (CIBERER), Hospital Virgen del Rocío, 41013 Sevilla, Spain; 5 HPC Service, University Information Services, University of Cambridge, Cambridge, UK; 6 Genotyping and Genetic Diagnosis Unit, Health Research Institute, INCLIVA, Valencia, Spain; 7 CIBERDem, Health Institute Carlos III, Madrid, Spain; 8 Institute for Integrative Systems Biology (I2SysBio), Universidad de Valencia-CSIC, 46980 Valencia, Paterna Spain; 9 Bioinformatics and Data Analysis Unit, Genomic Medicine Institute Imegen, Valencia, Spain; 10 Functional Genomics Node, INB-ELIXIR-es, Hospital Virgen del Rocío, 42013 Sevilla, Spain

**Keywords:** Gene therapy, Viral insertion, Viral integration, Sequence mapping, Genome viewer

## Abstract

**Background:**

The possibility of integrating viral vectors to become a persistent part of the host genome makes them a crucial element of clinical gene therapy. However, viral integration has associated risks, such as the unintentional activation of oncogenes that can result in cancer. Therefore, the analysis of integration sites of retroviral vectors is a crucial step in developing safer vectors for therapeutic use.

**Results:**

Here we present VISMapper, a vector integration site analysis web server, to analyze next-generation sequencing data for retroviral vector integration sites. VISMapper can be found at: http://vismapper.babelomics.org.

**Conclusions:**

Because it uses novel mapping algorithms VISMapper is remarkably faster than previous available programs. It also provides a useful graphical interface to analyze the integration sites found in the genomic context.

## Background

The stable, long-term correction of diseases by integrating viral vectors carrying healthy copies defective genes in the patient’s genome has become mainstream procedure in clinical gene therapy [1, 2]. However, despite its successful application, viral integration based therapies are not exempt of risks, such as the accidental activation of oncogenes that can cause malignant transformation of the cells [3, 4]. Vector locations in the host genome constitute molecular markers that help monitoring the fate of affected cells. Analysis of vector insertion sites (ISs) is carried out by the amplification (currently using Next Generation Sequencing –NGS- technologies) of sequences from retroviral vectors with a long terminal repeat (LTR). Primers mapping LTRs produce sequence reads with LTR-chromosome junctions, which can be used to accurately determine the chromosomal region of insertion of the viral vector [4]. Such monitoring is required because it is known that distinct gene transfer vectors can have preferences to target gene coding regions, CpG islands, or transcriptional start sites [5–7].

Here we present a new web server, VISMapper, a web tool to manage sequencing data for the detection of viral vector insertion sites in gene therapy experiments. VISMapper is much faster than other alternative software available and provides a comprehensive graphic interface that allows interactive visualization of the viral ISs in the genomic context.

## Implementation

VISMapper is written in Node.js (a JavaScript runtime) and uses GenomeMaps [8] for the visual representation of the results in the context of the genome. Thus the resulting viral insertion sites of an experiment can be visualized along with the genomic features they have around, including reads mapped, genes and other type of genomic elements. Supported assemblies for the human genome are GRCh37 and GRCh38.

Cancer genes were taken from the COSMIC [9] database through the CellBase [10] webservices.

## Results

### Data upload and workspace

VISMapper reads standard FASTQ or FASTA files containing reads corresponding to the insertion sites of the virus. If FASTA files are provided, they are converted to FASTQ format. Since FASTA files lack the quality parameter, this is set to 20 by default for the FASTQ file generated. A value of 20 minimizes the false positive rate when the original sequences are standard quality. In any case, the use of FASTQ containing quality values is obviously preferable. Files can be ZIP compressed. During the upload, user can optionally provide an email to be notified of the end of the data processing (given the speed of data processing it is usually unnecessary).

### Read mapping

Reads in the FASTQ file are mapped onto the reference human genome using BWA [11] or HPG-Align [12]. Typically mapping runtimes are in the range of seconds, which makes of VISMapper a truly interactive and accurate tool for exploring the result of retroviral insertion experiments. IS locations are detected by identified reads partially mapped. We use the CIGAR information for this. When the CIGAR of a mapping contains soft or hard clippings it indicates that the corresponding read have part of the genome sequence as well as part of the viral sequence. The reads are arranged by chromosome using SAMTools [13] and are inserted in a MySQL database for facilitating a faster access to them.

### Dashboard

**Fig. 1:**
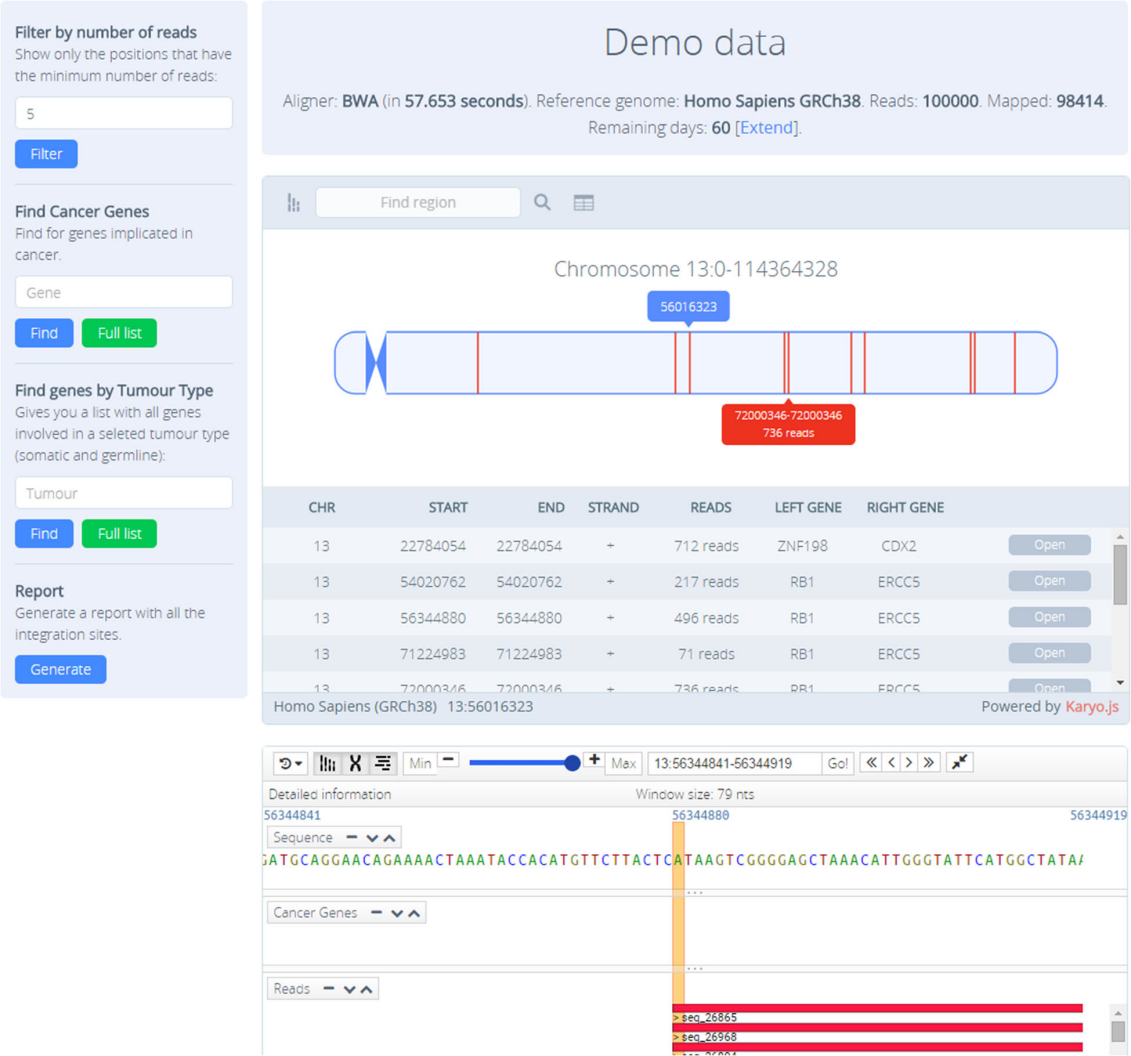
Screenshot showing the different graphical representations in the dashboard: the karyotype viewer and the genome viewer. Also, a table with the list of IS found is displayed

The Dashboard is a graphical working environment composed by three panels: the karyotype viewer, the genome viewer and the control panel (See Fig. 1). The karyotype viewer provides a general perspective of all the ISs along the chromosomes. Clicking with the left mouse button magnifies the chromosome, with ISs marked as red lines. Exact details on the IS location are provided by setting the cursor over them. A vertical panel on its left (See Fig. 1) allows filtering IS by the number of reads supporting them. It also allows searching those reads which are closer to oncogenes of genes related to specific tumor types. When the mouse hovers the chromosome in the karyotype a detailed view of the selected chromosome with the IS is displayed. Setting the mouse over the ISs pops up information on its exact location and the number of reads supporting it.

A more detailed view of the region in which the ISs occur (that can be selected by clicking in the karyotype viewer) can be obtained with the genome viewer, which implements GenomeMaps [8]. Several tracks are available at different detail level depending on the zoom level in the genome viewer: a) the surrounding genomic region, b) oncogenes located in the neighborhood (the cursor over them displays information on the genes) and c) reads mapped around the IS (again, information on the read, such as strand, mapping quality, etc. is provided by hovering the mouse on them).

Finally, the control panel allows setting a threshold based on the number of reads that support ISs and allows finding specific cancer genes or genes of specific cancer types (see Fig. 1, left part). Specifically, a box allows setting a threshold with the minimum number of reads to consider a IS (5 by default). The second box allows selecting a specific oncogene (can be searched by name or selected from a list). The list of oncogenes has been extracted from COSMIC. Another box allows displaying only the genes known to be associated with a given tumor.

### Report

The control panel allows generating a comprehensive tabular report of the results found. The button report directs to another page with a table containing all the ISs found that can be arranged by all the criteria shown in the header of the columns (chromosome, position, quality, etc.) Different filters (number of reads that support the IS and distance to a cancer gene) can be applied to expand or reduce the number of ISs to consider. This list can be downloaded in tab delimited format and a BAM file with the alignments found by the mapper can also be downloaded.

For any IS considered with the filtering schema used, the report contains the following items:

- Chromosome
- Position
- Number of reads mapped in this position
- Average quality of all the reads mapped in the position
- Closest oncogene
- Distance to the oncogene (0 means that the IS maps within the oncogene)
- Position of the oncogene with respect to the IS
- Entrez entry of the oncogene
- URL to the Entrez entry of the oncogene

### Comparison to other web servers for viral is mapping

**Fig. 2:**
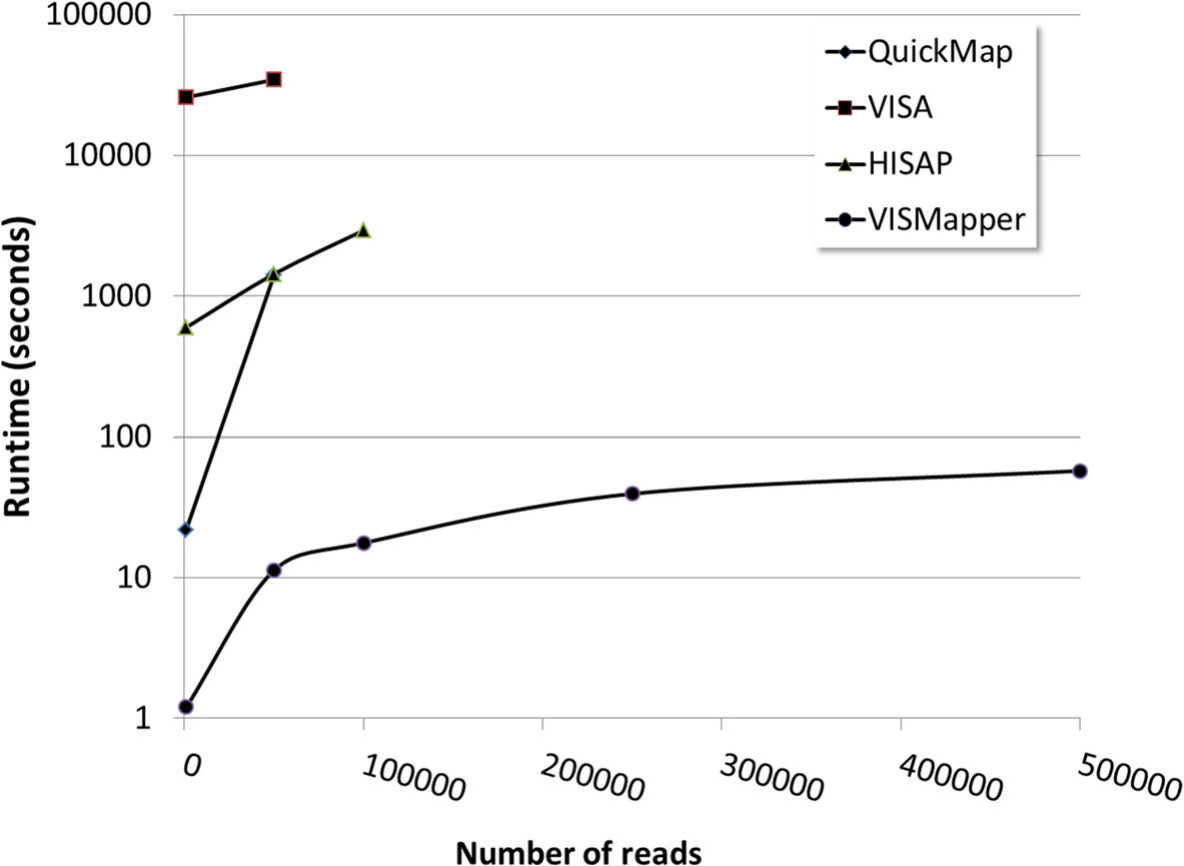
Runtimes observed for different programs QuickMap (line with diamonds), VISA (line with squares) HISAP (line with triangles) and VISMapper (line with circles) with datasets of increasing sizes. In the case of QuickMap, VISA and HISAP, the lines are interrupted according to internal hard limits for the number to sequences that the programs can process

There are a few web servers for viral vector insertion site analysis, such as, HISAP [14], SeqMap (requires user registration) or QuickMap [15], or the recently published VISA [16]. However, all of them use BLAST [17] or BLAT [18] for read mapping that involve comparatively much longer runtimes. Figure 2 shows a comparative of runtimes where the increase in speed gained by the use of more sophisticated mapping algorithms in VISMapper is obvious. The data used in the comparison were taken from the VISA website and can also be downloaded at the VISMapper documentation site (https://github.com/jmjuanes/vismapper/tree/master/ismapper-test).

In addition, a more detailed comparison was made with the VISA program by generating 4 datasets with known number of IS using the IS generator program from the VISA website (https://visa.pharmacy.wsu.edu/bioinformatics/random_site_generator.html). Table 1 shows the results of the comparison. Relative runtimes are similar to the ones shown in Fig. 2. While both methods give a very small number of false positives, in general VISMapper is able to map a higher percentage of sequences and found more IS sites than VISA.

**Table 1:** Comparison of VISA and VISMapper using four datasets generated with the IS generator program from the the VISMapper website (https://visa.pharmacy.wsu.edu/bioinformatics/random_site_generator.html)

Dataset	Input size (reads)	Insertion sites	Performance	VISA	VISMapper
Input 1			Runtime	~72 h	~60 s
	100,000	100,000	IS detected	99,694	99.793
			Total sequences mapped	99,694	99,881
Input 2			Runtime	~72 h	~60 s
	50,000	50,000	IS detected	49,854	49,897
			Total sequences mapped	49,855	49,936
Input 3			Runtime	~72 h	~30 s
	10,000	10,000	IS detected	9992	9969
			Total sequences mapped	9995	9981
Input 4			Runtime	~5 h	~30 s
	1000	1000	IS detected	906	929
			Total sequences mapped	906	930

Runtimes of both programs are shown for the four datasets, along with the number of sequences correctly mapped, that correspond to the IS detected, and the total number of sequences mapped, which in both cases is slightly superior, demonstrating a low rate of false positives in both cases

In addition, QuickMap does not process more than 50,000 sequences and VISA limits are between 50,000 and 100,000. HISAP could manage up to 100,000 in about 50 min, but cannot arrive to 250,000 sequences. Moreover, none of the other programs provide a graphic interface to analyze the results. Furthermore, QuickMap and HISAP do not support GRCh38.

## Conclusions

Because of its speed and sensitivity, VISMapper constitutes an attractive alternative to the options available for viral insertion site analysis. VISMapper offers a unique, interactive graphical working environment that allows a detailed and exhaustive exploration of the consequences and potential risks of the viral vectors inserted in the analyzed genome.

## Abbreviations

BAM: Binary alignment map
BWA: Burrows–wheeler algorithm
IS: Insertion Site
LTR: Long terminal repeat
NGS: Next generation sequencing
